# Taste Bud-Derived BDNF Is Required to Maintain Normal Amounts of Innervation to Adult Taste Buds^1^,^2^,^3^

**DOI:** 10.1523/ENEURO.0097-15.2015

**Published:** 2015-12-31

**Authors:** Lingbin Meng, Lisa Ohman-Gault, Liqun Ma, Robin F. Krimm

**Affiliations:** Department of Anatomical Sciences and Neurobiology, University of Louisville School of Medicine, Louisville, Kentucky 40292

**Keywords:** BDNF, geniculate ganglion, neurotrophins, plasticity, taste, taste bud

## Abstract

Gustatory neurons transmit chemical information from taste receptor cells, which reside in taste buds in the oral cavity, to the brain. As adult taste receptor cells are renewed at a constant rate, nerve fibers must reconnect with new taste receptor cells as they arise. Therefore, the maintenance of gustatory innervation to the taste bud is an active process. Understanding how this process is regulated is a fundamental concern of gustatory system biology. We speculated that because brain-derived neurotrophic factor (BDNF) is required for taste bud innervation during development, it might function to maintain innervation during adulthood. If so, taste buds should lose innervation when *Bdnf* is deleted in adult mice. To test this idea, we first removed *Bdnf* from all cells in adulthood using transgenic mice with inducible CreERT2 under the control of the Ubiquitin promoter. When *Bdnf* was removed, approximately one-half of the innervation to taste buds was lost, and taste buds became smaller because of the loss of taste bud cells. Individual taste buds varied in the amount of innervation each lost, and those that lost the most innervation also lost the most taste bud cells. We then tested the idea that that the taste bud was the source of this BDNF by reducing *Bdnf* levels specifically in the lingual epithelium and taste buds. Taste buds were confirmed as the source of BDNF regulating innervation. We conclude that BDNF expressed in taste receptor cells is required to maintain normal levels of innervation in adulthood.

## Significance Statement

Numerous correlative studies have suggested that neurotrophins are required to maintain peripheral sensory innervation in adulthood. However, this has not been tested in any peripheral sensory system. Because the taste receptor cells undergo constant renewal, nerve fibers continually reconnect to new taste receptors cells, making the maintenance of innervation to the taste bud an active process. Therefore, if any sensory system requires neurotrophins for its maintenance, it is likely to be the taste system. We show here that taste bud derived BDNF is required to maintain normal amounts of innervation to the taste bud in adulthood. This demonstrates that neurotrophins maintain sensory innervation. The requirement for BDNF in taste buds may be important for the tremendous plasticity of this system.

## Introduction

Taste receptor cells, which are organized into taste buds, detect the chemical content of food. Nerve fibers from gustatory neurons of the geniculate and petrosal ganglion innervate taste buds and carry taste information to the brain. A unique feature of taste receptor cells is that they have a limited lifespan and are constantly renewed (Beidler and Smallman, 1965; Perea-Martinez et al., 2013). As a result, gustatory neurons must continually locate and form functional connections with new adult taste receptor cells. Therefore, the maintenance of innervation to the taste bud is an active process, such that nerve fibers, which fail to seek out new taste receptor cells to innervate, will ultimately be lost. Given this plasticity, some mechanism/s must be present to direct nerve fibers to innervate taste receptor cells and function to maintain innervation over time.

Although we do not know how gustatory neurons identify and innervate new taste cells during adulthood, we do know how gustatory neurons innervate taste placodes during initial development. Taste placodes develop before the tongue is innervated and contain taste bud progenitor cells (Paulson et al., 1985; Thirumangalathu et al., 2009). Taste nerve fibers growing into the tongue are directed toward and locate developing taste placodes with little error (Mbiene and Mistretta, 1997; Lopez and Krimm, 2006a). Because this process requires that gustatory neurons identify a specific cell type (ie, taste placodal cells), the molecular mechanisms underlying taste bud innervation during development could provide clues as to how new taste receptor cells are innervated during adulthood.

The neurotrophin, brain-derived neurotrophic factor (BDNF) regulates initial innervation to the taste bud. Embryonically, BDNF is a neural attractant (Hoshino et al., 2010) specifically expressed in the placodes that will become taste buds (Nosrat et al., 1996, 2001; Huang and Krimm, 2010). BDNF overexpression in inappropriate regions misdirects innervation to incorrect locations in the lingual epithelium (Ringstedt et al., 1999; Krimm et al., 2001; Lopez and Krimm, 2006b). Conversely, gustatory axons fail to find and innervate taste placodes when BDNF is absent (Ma et al., 2009). Thus, BDNF is both necessary and sufficient for directing gustatory axons to specific targets. This role occurs during a critical period of gustatory development, after which BDNF is no longer required for targeting (Ma et al., 2009; Hoshino et al., 2010). However, BDNF continues to be expressed in taste buds throughout the lifespan (Yee et al., 2003). Interestingly, the pattern of BDNF expression changes during postnatal development. Over time, BDNF is downregulated from the gustatory precursor population, and as a result, becomes primarily expressed in a subpopulation of taste receptor cells (Huang et al., 2015). This places BDNF in a perfect location for maintaining innervation to a subpopulation of taste receptor cells during adulthood.

If BDNF is required for the active process of maintaining the innervation of taste receptor cells, then at least some innervation to taste buds should be lost after the removal of BDNF. The goal of the present study was to determine whether this was the case. Using inducible adult BDNF knock-out mice we demonstrate that BDNF is required to maintain normal levels of innervation in adulthood and that the source of this BDNF is the taste bud. We suggest that neurotrophins are particularly important for maintaining gustatory innervation in adulthood, because of this system’s tremendous plasticity.

## Materials and Methods

### Animals

To inducibly remove BDNF from all cells, we initially crossed mice expressing CreER under the control of the actin promoter (017595, Jackson Laboratories) or tamoxifen-inducible CreERT2 recombinase under the control of the Ubiquitin promoter (007001, Jackson Laboratories) with mice that express βGal when BDNF is removed (021055, Jackson Laboratories) to allow visualization of effective gene recombination (Gorski et al., 2003b). Experimental animals were produced by breeding these two Cre lines with mice in which exon 5 of the *Bdnf* gene is floxed (*Bdnf* ^lox/lox^; 004339, Jackson Laboratories). To increase the probability that *Bdnf* would be successfully removed, we removed *Bdnf* completely from one allele (*Bdnf* ^+/-^; 002266, Jackson Laboratories). After comparing gene recombination efficacy between the two Cre lines, we chose the CreERT2 mice as experimental animals. Therefore, the animals used for anatomical analysis were those that lacked a functional *Bdnf* gene in one allele and in which *Bdnf* could be inducibly removed from the other allele (CreERT2 *Bdnf* ^lox/-^). Three control genotypes were analyzed for three different purposes of comparison. *Bdnf* ^lox/+^ mice (with tamoxifen) were used to exclude potential effects of tamoxifen administration, and CreERT2 *Bdnf* ^lox/+^mice (without tamoxifen) were used to exclude the possibility of gene recombination in the absence of tamoxifen; both of these genotypes were expected to produce wild-type levels of *Bdnf*. Also, *Bdnf* ^lox^**^/-^**mice (with tamoxifen) were used to control for any effects of heterozygous *Bdnf* knockout.

To inducibly remove BDNF from the tongue epithelium, we crossed the same *Bdnf* floxed mice described above with mice expressing tamoxifen-inducible CreER recombinase under the control of a Keratin-14 promoter (K14-CreER; 005107, Jackson Laboratories). Gene recombination under the control of the K14-promoter has been shown to result in successful gene recombination in cells that become taste bud cells (Vasioukhin et al., 1999; Okubo et al., 2009). Experimental and control mice were the same as those described above. In addition, we bred K14-CreER mice with mice expressing tdtomato (007914) to visualize the effectiveness of tamoxifen-induced gene recombination.

### Tamoxifen administration

Mice were injected with tamoxifen (T5648, Sigma-Aldrich; mixed in peanut oil, 188 ng/g body weight) once per day for one (CreERT2 *Bdnf* ^lox/-^) or three (K14-Cre::CreERT2 *Bdnf* ^lox/-^) weeks by oral gavage. This dose has been used previously for effective inducible gene recombination in adult mice (Ruzankina et al., 2007; McGraw et al., 2011). When administered for more than 1 week, mice were given 2 d breaks between treatment sessions to recover from the drug. Tamoxifen injections were initiated in all mice between 2 and 2.5 months of age. CreERT2 mice were euthanized 2 or 10 weeks after final tamoxifen administration for real-time RT-PCR and 4 or 10 weeks after final tamoxifen administration for immunohistochemistry. K14-CreER mice were euthanized 10 weeks after final tamoxifen administration; one side of the tongue was used for RT-PCR, and the other side of the tongue was used for immunohistochemistry.

### Real-time RT-PCR

Mice were euthanized by overdose (4 mg/kg body weight) with avertin. The anterior tongue rostral to the circumvallate papilla was removed and rinsed with 0.1 m PBS solution, pH 7.4, and then cut in half evenly under a microscope. To isolate the lingual epithelium, tongue pieces containing fungiform papillae were incubated in sterile dispase I-solution (BD Biosciences) for 50–60 min. After incubation, epithelial sheets were peeled from the underlying lamina propria. The lingual epithelium from each mouse was stored in RNAlater stabilization solution (Ambion) and geniculate ganglia were stored in RNA isolation reagent (Qiagen) at 80°C until RNA extraction.

To determine the success of gene recombination, *Bdnf* mRNA levels in tongue epithelium and geniculate ganglia were measured using real-time RT-PCR. Total RNA from each geniculate ganglion and the epithelia was extracted using an RNeasy Micro Kit or RNeasy Mini Kit (Qiagen). DNase I treatment was applied to eliminate traces of DNA during the procedure. After extraction, RNA was analyzed with RNA 6000 Pico/Nano Chip Kits in a Bioanalyzer 2100 (Agilent Technologies), and RNA Integrity Number (RIN) and 28S:18S ratio were used to estimate RNA quality. Only RNA samples with an RIN >8.0 were used in this study. Reverse transcription was performed using 200 U Superscript III Reverse Transcriptase (Invitrogen) and 50 ng random hexamers (Invitrogen) in 25 ml reaction volume containing first strand buffer (Invitrogen), 0.5 mm dNTPs, and 40 U RNase inhibitor. All samples produced sufficient amounts of RNA for real-time RT-PCR. To control for differences in the amount of RNA isolated from different groups, the same amount of RNA was used from each geniculate ganglion (3 ng) and lingual epithelium (50 ng) sample. After incubation for 50 min at 50°C, the reaction was stopped by heating (5 min at 85°C).

Real-time RT-PCR was performed with the ABI PRISM/7900HT sequence detection system (Applied Biosystems) using the Taq-Man Universal PCR Kit (Applied Biosystems) and oligonucleotide primer/probe sets, which were designed from sequences in the GenBank database using Beacon Designer software (Premier Biosoft International). When possible, primers were chosen to span an intron to avoid any genomic DNA contamination. TaqMan probes were labeled at the 5′-end with a fluorescent reporter dye (fluorescein; FAM) and at the 3′-end with a quencher dye (carboxytetramethylrhodamine; TAMRA). Real-time RT-PCR reactions (Table 1) were conducted using 10 µl total volume, with Master Mix, 720/200 nm primer/probe sets (TaqMan PCR Kit), and the same amount of target cDNA across different time periods. For normalization of cDNA loading, all samples were run in parallel with the housekeeping genes 18S ribosomal RNA and mouse glyceraldehyde 3 phosphate dehydrogenase (GAPDH). Each assay was carried out in triplicate. Amplification of cDNA was performed for 40 cycles at 95°C for 15 s and 60°C for 1 min.

**Table 1.** Sequences of primer pairs and probes used for real-time RT-PCR

Gene GenBack accession no.		Sequence 5′–3′	Fragment size, bp
*Bdnf*	(X55573)		110
	Forward primer	TGCAGGGGCATAGACAAAAGG	
	Reverse primer	CTTATGAATCGCCAGCCAATTCTC	
	Taqman Probe	ACTGGAACTCGCAATGCCGAACTACCCA	
*Ntf4*	(NM[lowem]198190)	95	
	Forward primer	AGCGTTGCCTAGGAATACAGC	
	Reverse primer	GGTCATGTTGGATGGGAGGTATC	
	Taqman Probe	TGAGCAGTGAACCCGACCACCCAGG	
*Ntf3*	(NM-001164034)		
	Forward primer	CAGAACATAAGAGTCACCGGAA	94
	Reverse primer	TGTCCCCGAATGTCAATGG	
	Taqman Probe	CACCCACAGGCTCTCACTGTC	
*TrkB*	(X17647)		86
	Forward primer	AAGGACTTTCATCGGGAAGCTG	
	Reverse primer	TCGCCCTCCACACAGACAC	
	Taqman Probe	CCAACCTCCAGCACGAGCACATTGTCAA	
*P2rx3*	(MN-145526)		113
	Forward primer	TTTCCCCTGGCTACAACTTC	
	Reverse primer	CCCGTATACCAGCACATCAAAG	
	Taqman Probe	AGA TGG AGA ATG GCA GCG AGT ACC G	
18S rRNA	(X00686)	CAGGATTGACAGATTGATAGCTCTTTC	76
	Forward primer	ATCGCTCCACCAACTAAGAACG	
	Reverse primer	CCATGCACCACCACCCACGGAATCG	
	Taqman Probe	130	
GAPDH	(NM_008084)	AATGTGTCCGTCGTGGATCTG	
	Forward primer	CAACCTGGTCCTCAGTGTAGC	
	Reverse primer	CGTGCCGCCTGGAGAAACCTGCC	
	Taqman Probe		
β-Actin	(NM_007393)	CTGGGACGACATGGAGAAGATC	144
	Forward primer	GTCTCAAACATGATCTGGGTCATC	
	Reverse primer	ACCTTCTACAATGAGCTGCGTGTGGCC	
	Taqman Probe		

### Immunohistochemistry

Mice were euthanized by avertin overdose (4 mg/kg body weight), perfused through the heart using 4% paraformaldehyde (PFA), and post fixed in PFA for 2 h or immersion-fixed in 4% PFA overnight. Geniculate ganglia were dissected under a microscope. Tissues were then rinsed with PBS and transferred to 30% sucrose at 4°C overnight. Tissues were frozen the next day in OCT and stored at −80°C until sectioned on a cryostat. Serial sagittal sections of the tongue (20 or 70 µm) and geniculate ganglion (50 µm) were mounted on glass slides.

To visualize taste buds and innervation in serial thin sections, slides containing tongue sections were first dried on a slide warmer (37°C) overnight. The next day, they were rehydrated, placed into citric acid buffer (10 mm citric acid, 0.05% Tween 20, pH 6.0), heated to 98°–100°C for ∼15 min in a boiling water bath, and allowed to cool for 20 min at room temperature for antigen retrieval. Slides were washed in PBS, pH 7.4, and incubated overnight at room temperature in anti-cytokeratin-8 antibody in PBS (Deveopmental studies Hybridoma Bank, Troma-1-s). Following incubation with primary antibody, slides were rinsed in PBS and incubated for 1.5 hours with AlexaFluor 488 anti-rat secondary antibody (1:500; Invitrogen). After washing in PBS, slides were incubated for 2 h in blocking solution (5% normal goat serum and 2% Triton X-100 in PBS) followed by rabbit anti-P2X3 (1:500; Millipore, AB5895) and mouse anti-TUJ1 (1:500; Covance, MMS-435P) antibodies in blocking solution at room temperature overnight. After overnight incubation with primary antibodies, slides were rinsed in PBS (4 times for 5 min) and incubated for 1.5 h at room temperature in AlexaFluor 555 anti-rabbit (1:500; Invitrogen) and AlexaFluor 647 anti-mouse (1:500; Invitrogen) secondary antibodies in blocking solution. Slides were then rinsed in PBS (3 times for 5 min) and stained with DAPI (4,6-diamidino-2-phenylindole dihydrochloride; 2 µl in 50 ml double-distilled H_2_O; Life Technologies) for 15 min. After rinsing in PBS (3 times for 5 min), slides were mounted with fluoromount-G (SouthernBiotech).

To visualize taste buds and innervation in thick floating sections, tongues were removed fresh; one side was processed for RT-PCR and the other side was immersion-fixed overnight, cryoprotected overnight, and then frozen for sectioning. Floating sections (70 µm) were incubated with primary antibodies for 7 d, rinsed for 4 h, incubated in secondary antibodies for 2 d, rinsed for 4 h, and then mounted. Primary antibodies included anti-cytokeratin-8, anti-P2X3 (concentrations and companies were the same as described above). Slides were coded after immunohistochemistry such that all analyses were conducted by an experimenter who was blind to mouse genotype and tamoxifen treatment.

To quantify taste receptor cell types in whole taste buds, the tongue epithelium was separated from the muscle with scissors and then flattened and frozen in OCT. More muscle is removed using a cryostat such that only a thin layer of muscle remains attached to the epithelium. The tongue epithelium was then processed for whole mount immunohistochemistry using the same protocol as for thick sections. Primary antibodies were goat anti-Car4 (R&D Systems, AF2414, 1:500), rabbit anti-PLCβ2 (Santa Cruz Biotechnology, sc-206, 1:500), and rat anti-cytokeratin 8, and secondary antibodies included AlexaFluor 647 anti-goat, AlexaFluor 555 anti-rabbit, and AlexaFluor 488 anti-rat. The tissues were counter stained with DAPI and mounted with the epithelium side up.

### Taste bud counts

Taste buds were defined with cytokeratin-8 staining and by their location in fungiform papillae. Taste buds were counted in 20 µm serial sagittal sections of the tongue using a Leica DMLB microscope. The entire taste bud was followed through each section so that taste buds were only counted once.

### Stereology

Stereological methods were used to count the total number of geniculate ganglion neurons. Fixed ganglia were serial sectioned (50 μm), mounted on slides, and stained with cresyl violet for 15 min. To maintain section thickness, sections were not dehydrated and were mounted in Dako glycergel mounting medium (Dako North America) for stereological quantification. Using StereoInvestigator software (MBF Bioscience), an experimenter blind to mouse genotype traced a contour around the geniculate ganglion (20× magnification). Every section containing the ganglion was traced, and at least four sections were quantified. Within each traced contour, the computer randomly determined the placement of the counting frames. The depth of the counting frame was equal to the minimal thickness of the section minus a guard zone of 10 μm (ie, 5 μm from the top and bottom of the section). Geniculate ganglion neurons were counted (100× magnification) in each counting frame (15 μm^2^). Neurons were counted only when they first came into focus (cell top) so that each cell was counted only once. Based on these measurements, total cell number in each ganglion was estimated for the entire volume of the ganglion using the optical fractionator probe (MBF Bioscience).

To measure cell diameter in these same ganglia, we measured cell size by capturing four images of each ganglion no images were captured from adjacent sections. These images were imported into ImageJ software where the areas of 100 neuron cell bodies were measured per ganglion. To avoid measuring split cell profiles, only neurons with a clear nucleus were measured. Diameters were calculated from the areas measured for each cell.

### Quantification of taste bud size, innervation, and number of taste cells in thin sections

**Figure 1.**
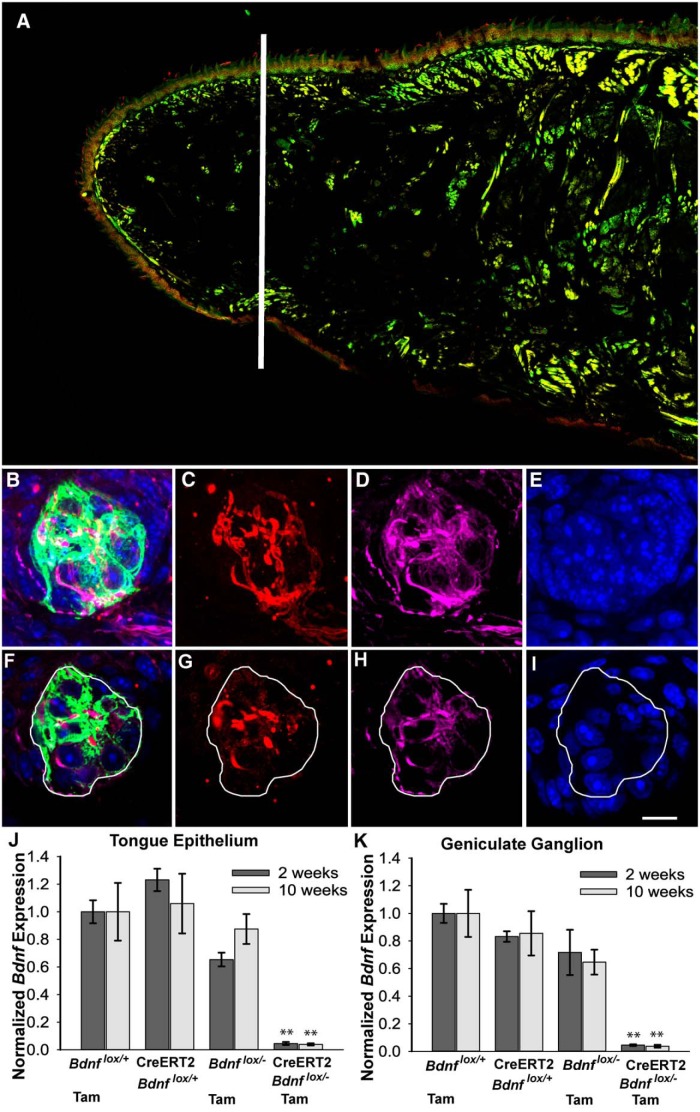
Taste bud size, innervation, and cell number were quantified in adult mice after *Bdnf* gene recombination. Because taste buds are larger at the back than at the front of the tongue, only taste buds from the tongue tip, shown as the region anterior to the line in ***A***, were imaged. Z-stacked images of a representative taste bud showing (***B***) all four labels (anti-cytokeratin-8; green), (***C***) P2X3 (red), (***D***) anti-TUJ1 (magenta), and (***E***) DAPI (blue). ***F***–***I***, Single optical sections from the z-stack. ***B***, Cytokeratin-8 labeling (green) was used to define the border of taste buds, and the number of (***G***) red and (***H***) magenta pixels determined the volume of P2X3- and TUJ1-positive innervation, respectively, within taste buds. ***I***, Nuclei stained with DAPI (blue) were followed through optical sections so that they were counted only once. Three control genotypes (*Bdnf* ^lox/+^, CreERT2 *Bdnf* ^lox/+^, *Bdnf* ^lox/-^) had normal levels of *Bdnf* expression in the (***J***) tongue epithelium and (***K***) geniculate ganglion 2 and 10 weeks after tamoxifen (Tam) administration. Expression level is normalized to *Bdnf* ^lox/+^ mice. However, CreERT2 *Bdnf* ^lox/-^ mice showed reduced *Bdnf* expression in the (***J***) tongue epithelium and (***K***) geniculate ganglion 2 and 10 weeks after tamoxifen administration, indicating effective gene recombination in these mice. Scale bar: (in ***I***) ***B***–***I***, 10 µm. **p ≤ 0.01.

Individual taste buds at the tip of the tongue (Fig. 1*A*) were imaged for subsequent analysis, thereby reducing anterior-posterior variation in taste bud size, using an Olympus Fluoview FV1000 Laser scanning confocal microscope (LSI3-FV1000-Inverted). During both image capture and analysis, the experimenter was blind to mouse genotype. For each optical image, the four channels were collected separately with single wavelength excitation and then merged to produce a composite image. Serial optical sections were captured every 1 μm in labeled whole taste buds at 60× magnification, 3.5 zoom. As a single taste bud from an intact tongue was typically found in two to three physical sections (ie, 36–60 μm), all sections containing the taste bud were captured so that the entire taste bud could be quantified. Each physical section contained 15–20 optical sections. Each 1 μm optical section was traced (Fig. 1*F*–*I*), and the traced area was measured using Neurolucida imaging software (MicroBrightField). The area for each 1 μm optical section was summed across all physical sections to yield taste bud volume. The volume of anti-P2X3 and anti-TUJ1 immunostaining in taste buds was measured using MBF ImageJ software (v1.47; Jensen, 2013), which set an unbiased threshold automatically, and pixels were analyzed for every section. Finally, the number of labeled pixels inside a taste bud was counted for each section and summed to obtain the total volume of nerve fibers for each taste bud.

Taste cell number was quantified by counting DAPI staining within keratin 8-defined taste buds. DAPI-labeled nuclei were followed through the optical sections such that each nucleus was only counted once. Because each taste bud appeared in more than one physical section, the number of cells was added for each physical section. A few cells may have been counted twice due to split nuclei.

### Quantification of taste bud size, innervation, total number of taste cells, and number of taste receptor cells in thick sections and whole mounts

All taste buds on the front two-thirds of the tongue were imaged using an Olympus confocal microscope with an optical Z-stack thickness of 0.47 µm. Taste bud files were coded and analyzed blind to mouse genotype. Imaris software (Bitplane, http://www.bitplane.com/contact) was used to first rotate the taste bud and determine whether the whole taste bud had been captured; all whole taste buds for each mouse were analyzed. The volume was determined by outlining the taste bud as defined by cytokeratin-8 labeling in each optical section. This outline determined the surface area of a taste bud and was used to generate a 3D surface. The volume within this surface was calculated by the software and represents taste bud volume. The masking feature available in Imaris was used to mask all signals from the 546 channel (representative of P2X3-labeled nerve fibers) within the boundaries of the surface. This mask included red signal only within the taste bud and excluded red signal outside the taste bud surface. A surface of P2X3 innervation within the taste bud was created based on this mask, and the volume within this surface was calculated by the software to measure total innervation within the taste bud. This analysis was conducted by an experimenter who was blind to mouse genotype and tamoxifen treatment.

Taste buds from the tongue tip were imaged on an Olympus confocal microscope with an optical Z-stack thickness of 1 µm. Because this tissue was processed as whole mounts, images were collected in cross-section from the base of the taste bud to the taste pore. These files were analyzed in Stereo-investigator; individual taste cells were followed through the confocal image stack so that each cell was only counted once. In a single section containing the nucleus, the cell was defined as Car4-positive, or PLCβ2-positive. Keratin 8-positive cells were also counted. The number of labeled cells of each type was collected for seven taste buds/animal and averaged.

### Statistical analysis

All measurements within the same group of mice were determined to have equal variance (using Levene’s test for homogeneity of variance, *p* > 0.05). For *Bdnf* mRNA levels, taste bud volume, nerve innervation, taste cell number, taste bud number, and ganglion cell number, two-way ANOVAs were used. After overall significance was determined, Tukey *post hoc* tests were used to identify significant differences in pairwise comparisons when differences were found across genotype (Table 2). The mean value of each group represented four to five different mice. For analysis of individual taste buds, the mean value of each mouse represented five to seven different taste buds. Five CreERT2 *Bdnf* ^lox/^**^-^**mice and four mice from each of the other genotypes (*Bdnf* ^lox/+^, CreERT2 *Bdnf* ^lox/+^, and *Bdnf* ^lox/+^) were analyzed. One-way ANOVA followed by Tukey’s posthoc tests were used to compare taste bud volume and amount of innervation in a second set of mice with the following genotypes: K14-CreER *Bdnf* ^lox/-^, *Bdnf* ^lox/+^, K14-CreER *Bdnf* ^lox/+^, and *Bdnf* ^lox/+^ (*n* = 4/genotype; Table 2). For taste cell types the two genotypes (*n* = 3) were compared with a *t* test. The statistical significance level was set at *p* < 0.05 for all comparisons.

**Table 2.** Statistical table

	Measure	Data structure	Type of test	Power
**a (** Fig. 1J)	*Bdnf* expression, epithelium	Normally distributed	2 X 4 ANOVA, Tukey *post hoc*	0.999
**b (** Fig.1K**)**	*Bdnf* expression, ganglion	Normally distributed	2 X 4 ANOVA, Tukey *post hoc*	0.995
**c**	Body weight	Normally distributed	2 X 4 ANOVA	0.999
**d**	*Ntf3*- expression, epithelium	Normally distributed	one-way ANOVA	0.625
**e**	*Ntf4*-expression, epithelium	Normally distributed	one-way ANOVA	0.36
**f (** Fig. 2A**)**	Geniculate ganglion number	Normally distributed	2 X 4 ANOVA	0.876
**g (** Fig. 2B**)**	Taste bud number	Normally distributed	2 X 4 ANOVA	0.891
**h. (** Fig 2C**)**	Geniculate ganglion neuron size	Normally distributed	one- way ANOVA	0.985
**i (** Fig. 3Q**)**	P2X3 label volume	Normally distributed	2 X 4 ANOVA, Tukey *post hoc*	0.999
**j**	P2X3 expression	Normally distributed	one-way ANOVA	0.642
**k (** Fig. 3R**)**	Tuj1 label volume	Normally distributed	2 X 4 ANOVA, Tukey *post hoc*	0.99
**l (** Fig. 4E**)**	Taste bud volume	Normally distributed	2 X 4 ANOVA, Tukey *post hoc*	0.95
**m (** Fig 4F**)**	No. of cells/bud	Normally distributed	2 X 4 ANOVA, Tukey *post hoc*	0.95
**n**	PLCβ2-cell number	Normally distributed	t-test	0.636
**o**	Car4-positive taste cell number	Normally distributed	t-test	0.844
**p**	Cytokeratin-8- positive taste cell number	Normally distributed	t-test	0.966
**q (** Fig. 6A**)**	P2X3-label X taste bud volume	*X*,*Y*	Pearson product-moment correlation	0.999
**r (** Fig. 6B**)**	P2X3-label X taste bud cell number	*X*,*Y*	Pearson product-moment correlation	0.999
**s (** Fig. 6C**)**	TUJ1-label X taste bud volume	*X*,*Y*	Pearson product-moment correlation	0.999
**t (** Fig. 6D**)**	TUJ1-label X taste cell number	*X*,*Y*	Pearson product-moment correlation	0.999
**u (** Fig. 7F**)**	*Bdnf*-expression	Normally distributed	one-way ANOVA	0.995
**v (** Fig 8G**)**	P2X3-label volume	Normally distributed	one-way ANOVA	0.97
**w (** Fig. 8H**)**	Taste bud volume	Normally distributed	one-way ANOVA	0.153

## Results

### *Bdnf* expression is reduced in adult mice with *Bdnf* gene deletion

In the mouse taste system, BDNF continues to be expressed during postnatal development and adulthood (Yee et al., 2003). To study its function, we needed to effectively eliminate *Bdnf* expression in adult mice without influencing their development. Because *Bdnf* is expressed in taste buds, the geniculate ganglion, and the CNS, all of which could influence taste neurons, we began by inducibly removing *Bdnf* from all cells using a ubiquitously express promoter. When we compared CAGGS-CreER::*Bdnf* ^loxlacZ/+^ (Hayashi and McMahon, 2002) mice with CreERT2:: *Bdnf* ^loxlacZ/+^mice, (Cre recombinase expression is under the control of an ubiquitin promoter) we found that both constructs yield βgal expression in all taste buds, even at low doses of tamoxifen. However, CreER *Bdnf* ^lox/-^ mice were more effective at reducing BDNF mRNA levels than CAGGS-CreER mice. Therefore, we decided to use CreER *Bdnf* ^lox/-^ mice transgenic mice because they allow effective removal of genes after 1 week of tamoxifen administration in adult mice (Ruzankina et al., 2007).

Following a week of tamoxifen administration initiated when mice were 2–2.5 months of age, we found that *Bdnf* expression was reduced dramatically in the tongue epithelium and geniculate ganglion of CreERT2 *Bdnf* ^lox/-^ mice compared with three control genotypes (*Bdnf* ^lox/+^, *p* < 0.001; CreERT2 *Bdnf* ^lox/+^, *p* < 0.001; *Bdnf* ^lox/-^, *p* = 0.001). *Bdnf* expression was measured 2 weeks after the end of tamoxifen administration (Fig. 1*J*,*K*). To verify that *Bdnf* expression remained low throughout the experiment, we also examined *Bdnf* expression 10 weeks after tamoxifen administration. Most CreERT2 *Bdnf* ^lox/-^ mice (3 of 4) still showed a dramatic reduction of *Bdnf* expression in the tongue epithelium and geniculate ganglion compared with control mice (*Bdnf* ^lox/+^, *p* < 0.05 for epithelium and *p* < 0.005 for ganglion; CreERT2 *Bdnf* ^lox/+^, *p* < 0.01 for both epithelium and ganglion; *Bdnf* ^lox/-^, *p* < 0.05 for both epithelium and ganglion; Fig. 1*J*,*K*) 10 weeks after tamoxifen administration. There was no effect of tamoxifen administration alone on *Bdnf* expression (*Bdnf* ^lox/+^ mice with tamoxifen vs. CreERT2 *Bdnf* ^lox/+^ mice without tamoxifen). Moreover, heterozygous *Bdnf* mutant mice (*Bdnf* ^lox/-^) had similar *Bdnf* expression levels as *Bdnf* ^lox/+^ and CreERT2 *Bdnf* ^lox/+^ mice, indicating that one functional *Bdnf* allele produces as much *Bdnf* mRNA as two alleles. Together, these findings demonstrate that *Bdnf* expression is successfully reduced to 5% of normal levels in the tongue epithelium and 4% of normal levels in the geniculate ganglion in most experimental mice after tamoxifen administration. Furthermore, *Bdnf* expression remained low for the duration of the experiment.

Deletion of *Bdnf* from the hypothalamus of adult mice results in hyperphagic behavior and obesity (Lyons et al., 1999; Unger et al., 2007). Because the ubiquitin promoter is expressed in all cells, weight gain in CreERT2 *Bdnf* ^lox/-^ mice after tamoxifen administration could be considered evidence of effective gene recombination. Indeed, CreERT2 *Bdnf* ^lox/-^ mice appeared to be heavier than their littermate control mice. Before tamoxifen administration, the average body weight of all four genotypes of mice was 20 g, with no difference among genotypes (data not shown). In contrast, the CreERT2 *Bdnf* ^lox/-^ mice had an average body weight of 42 g 4 weeks after tamoxifen administration, whereas the other three control genotypes (*Bdnf* ^lox/+^, *p* < 0.001; CreERT2 *Bdnf* ^lox/+^, *p* < 0.001; *Bdnf*^lox/-^, *p* < 0.001) had an average body weight of 24 g. Ten weeks after tamoxifen administration, CreERT2 *Bdnf* ^lox/-^ mice had an average body weight of 50 g, whereas the other three control genotypes had an average body weight of 30 g (*Bdnf* ^lox/+^, *p* < 0.001; CreERT2 *Bdnf* ^lox/+^, *p* < 0.001; *Bdnf* ^lox/-^, *p* < 0.001). In addition to weight gain, we noticed increased circling behavior in CreERT2 *Bdnf* ^lox/-^mice, which could be related to vestibular and/or cerebellar dysfunction.

Both BDNF and neurotrophin (NT)-4 function through a common TrkB receptor (Barbacid, 1994; Naylor et al., 2002; Huang and Reichardt, 2003). NT-3 primarily binds and activates TrkC; however, NT-3 may also function through TrkA and TrkB receptors (Fariñas et al., 1998). Therefore, the expression of these factors could increase to compensate for the absence of BDNF after *Bdnf* gene deletion. To test this idea, we measured the expression of these neurotrophic factors in the lingual epithelium 10 weeks after tamoxifen administration. There were no differences across genotypes in expression of *Ntf3* (normalized expression level, mean ± SE; CreERT2 *Bdnf* ^lox/^, 0.77 ± 0.08; *Bdnf* ^lox/+^, 1.00 ± 0.15; CreERT2 *Bdnf* ^lox/+^, 0.78 ± 0.09; *Bdnf* ^lox/-^, 0.97 ± 0.12) or *Ntf4* (CreERT2 *Bdnf* ^lox/-^, 0.87 ± 0.16; *Bdnf* ^lox/+^, 1.00 ± 0.13; CreERT2 *Bdnf* ^lox/+^, 0.78 ± 0.09; *Bdnf* ^lox/-^, 0.97 ± 0.13) within the tongue epithelium. Therefore, *Ntf3* and *Ntf4* expression did not increase to compensate for reduced *Bdnf* expression.

### Geniculate ganglion neuron and taste bud number are unaffected by *Bdnf* gene deletion

**Figure 2.**
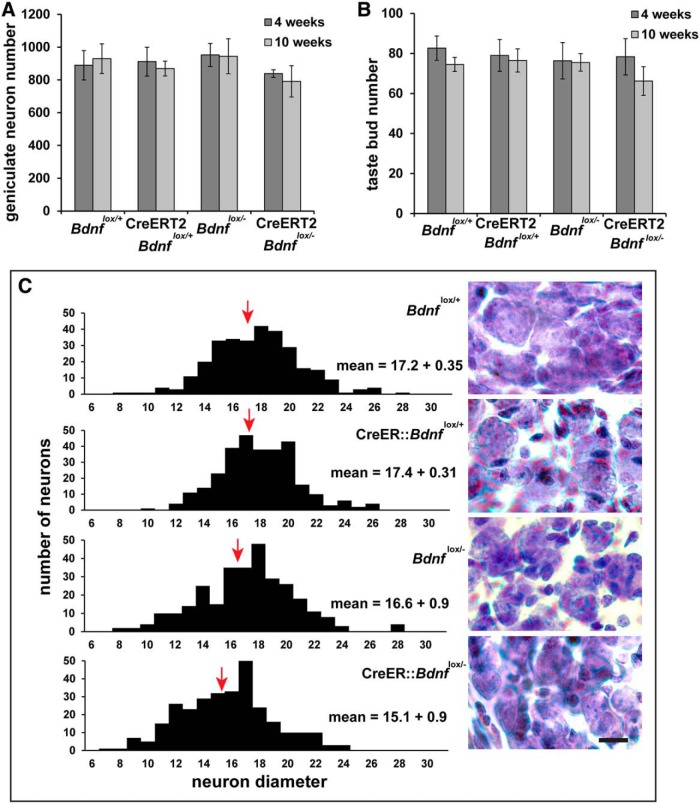
Taste bud and geniculate ganglion numbers were maintained, but neurons were smaller after *Bdnf* gene removal during adulthood. ***A***, Geniculate ganglion neuron number and (***B***) taste bud number were unchanged 4 and 10 weeks after tamoxifen (Tam) administration. ***C***, We measured the diameters of 100 neurons per animal from images taken of each ganglion. Histograms show the distributions of neuron sizes, with the median indicated (red arrow). We also averaged these values in each animal (*n* = 3) and compared the means of these data points using an ANOVA. Means and standard errors are also shown on the Figure. Scale bar, 10 µm.

BDNF is required for the survival of gustatory neurons and the maintenance of taste buds during development (Nosrat et al., 1997; Zhang et al., 1997; Mistretta et al., 1999; Patel et al., 2010). However, whether BDNF is also required to support geniculate neuron survival and/or maintain taste bud number in adulthood is unknown. To answer these questions, we counted the number of geniculate neurons and taste buds on the anterior two-thirds of the tongue containing the fungiform taste field. Geniculate ganglion neuron number (Fig. 2*A*) and taste bud number (Fig. 2*B*) were not different between CreERT2 *Bdnf* ^lox/-^ mice and mice with normal *Bdnf* expression levels (*Bdnf* ^lox/+^, CreERT2 *Bdnf* ^lox/+^, and *Bdnf* ^lox/-^) at 4 and 10 weeks after tamoxifen administration. Therefore, neither geniculate ganglion neuron number nor taste bud number were regulated by BDNF deletion during adulthood.

Blocking NGF function in adult animals reduces neuron cell body size, even though neuron number is largely unaffected (Angeletti et al., 1971). To determine whether neuron cell body size was reduced following BDNF-removal, we measured the diameter of 100 neurons per ganglion for each animal. We found a reduction in cell soma size with BDNF removal, which can be seen as a shift in the histograms and median value for cell size across genotypes (Fig. 2*C*, red arrow). Mean geniculate neuron diameters were reduced in the experimental (CreERT2 *Bdnf* ^lox/-^ mice) compared with the three control genotypes (*Bdnf* ^lox/+^, CreERT2 *Bdnf* ^lox/+^, and *Bdnf* ^lox/-^; *p* ≤ 0.02).

### Neural innervation of taste buds is reduced by half after *Bdnf* gene deletion

**Figure 3.**
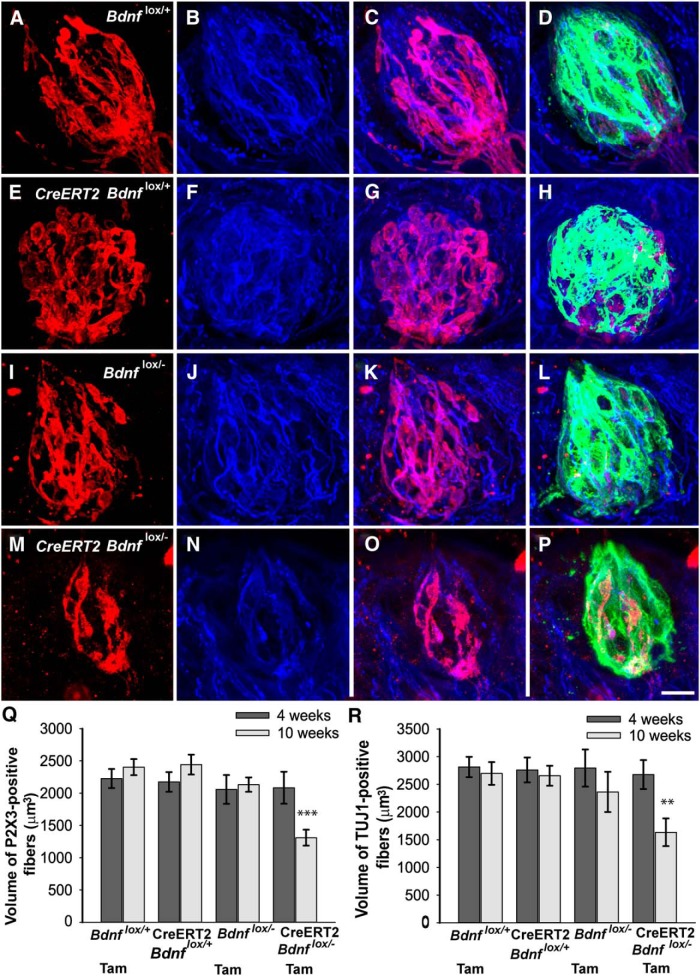
The volume of innervation within taste buds was reduced after *Bdnf* gene removal during adulthood. Ten weeks after tamoxifen administration, taste buds in (***A***–***D***) *Bdnf* ^lox/+^, (***E***–***H***) CreERT2 *Bdnf* ^lox/+^, and (***I***–***L***) *Bdnf* ^lox/-^ mice appeared to be roughly the same size and have similar amounts of (***A***, ***E***, ***I***) P2X3-positive and (***B***, ***F***, ***J***) TUJ1-positive innervation. However, taste buds in (***M***–***P***) CreERT2 *Bdnf* ^lox/+^ mice appeared smaller and to have less (***M***) P2X3-positive and (***N***) TUJ1-positive innervation. ***Q***, P2X3-positive and (***R***) TUJ1-positive innervation to taste buds was reduced 10 weeks but not 4 weeks after tamoxifen (Tam) administration. Scale bar: (in ***P***) ***A***–***P***, 10 µm. **p ≤ 0.01, ***p ≤ 0.001.

During development, BDNF is required for innervation of the gustatory epithelium (Ringstedt et al., 1999; Lopez and Krimm, 2006b; Ma et al., 2009). However, whether neurotrophins are required for maintaining the proper amount of gustatory innervation during adulthood is unclear. To answer this question, we labeled nerve fibers using two markers, anti-P2X3 (red) and anti-TUJ1 (blue), to analyze innervation within taste buds defined by cytokeratin-8 (green; Fig. 3*A*–*P*). P2X3 is an ATP channel required for neural responses to taste stimuli (Finger et al., 2005; Murata et al., 2010; Taruno et al., 2013) and, in the tongue, is specific to taste fibers originating from the geniculate ganglion (Finger et al., 2005; Ishida et al., 2009). TUJ1 (anti-neuron specific beta III tubulin) is a general marker of nerve innervation. Both antibodies clearly labeled nerve fibers within taste buds, although the labels were overlapping in some locations and distinct in others (Fig. 3*C*,*G*,*K*,*O*). No obvious differences were observed among the four genotypes in the amount of P2X3 or TUJ1 staining within taste buds 4 weeks after tamoxifen administration (images not shown). However, 10 weeks after tamoxifen administration, CreERT2 *Bdnf* ^lox/-^ mice appeared to have fewer labeled nerve fibers and smaller taste buds than the other three genotypes (Fig. 3*M*–*O*).

To quantify these observations, we analyzed the volume of P2X3- and TUJ1-positive nerve fibers inside taste buds. Four weeks after tamoxifen administration, mice with reduced levels of *Bdnf* expression (CreERT2 *Bdnf* ^lox/-^) had similar volumes of P2X3 staining (Fig. 3*Q*) and TUJI staining (Fig. 3*R*) inside taste buds compared with mice with normal levels of *Bdnf* (*Bdnf* ^lox/+^, CreERT2 *Bdnf* ^lox/+^, and *Bdnf* ^lox/-^). There were also no differences in the volume of P2X3- or TUJ1-labeled fibers among the three control genotypes, demonstrating that neither tamoxifen nor elimination of a single *Bdnf* allele changed the amount of taste bud innervation. However, 10 weeks after tamoxifen administration, CreERT2 *Bdnf* ^lox/-^ mice showed significantly less P2X3 staining compared with the control genotypes (*Bdnf* ^lox/+^, *p* < 0.001; CreERT2 *Bdnf* ^lox/+^, *p* < 0.001; *Bdnf* ^lox/-^, *p* < 0.001; Fig. 3*Q*). Similarly, CreERT2 *Bdnf* ^lox/-^ mice showed less TUJ1 staining compared with the control genotypes (*Bdnf* ^lox/+^, *p* < 0.01; CreERT2 *Bdnf* ^lox/+^, *p* < 0.01; *Bdnf* ^lox/-^, *p* < 0.05) 10 weeks after tamoxifen administration (Fig. 3*R*). Again, no differences among the three control genotypes were observed at 10 weeks, confirming that neither tamoxifen administration nor a single *Bdnf* allele influences taste bud innervation. Therefore, *Bdnf* appears to be required for the long-term maintenance of normal P2X3-positive and TUJ1-positive taste bud innervation during adulthood.

Unlike expression of TUJ1, which is a structural protein, expression of P2X3 is frequently altered by experimental manipulation (Banerjee et al., 2009; Zhang et al., 2014; Su et al., 2015). To verify that *Bdnf* gene deletion specifically affected innervation and not simply P2X3 expression, we used real-time RT-PCR to detect P2X3 expression in the geniculate ganglion 10 weeks after tamoxifen administration. There were no differences in P2X3 expression among genotypes (CreERT2 *Bdnf* ^lox/-^, 0.78 ± 0.08; *Bdnf* ^lox/+^, 1.00 ± 0.17; CreERT2 *Bdnf* ^lox/+^, 1.09 ± 0.13; *Bdnf* ^lox/-^, 0.92 ± 0.13). Therefore, P2X3 expression was not influenced by *Bdnf* gene deletion, and the loss of P2X3-positive fibers did not reflect merely a reduction in P2X3 expression.

As there are no perfect labels for taste bud innervation, we used two different markers of nerve fibers (P2X3 and TUJ1) and limited our analysis to taste buds. Because some labeling was non-overlapping, we compared the two markers to determine whether changes in one label predicted changes in the other label. In general, individual taste buds containing more P2X3-stained fibers also contained more TUJ1-stained fibers (*r* = 0.822, *p* < 0.001, *n* = 81). Because the two different markers of nerve innervation produced similar results, we conclude that BDNF is required to maintain normal levels of innervation to taste buds during adulthood.

### Changes in innervation predict changes in taste bud size

**Figure 4.**
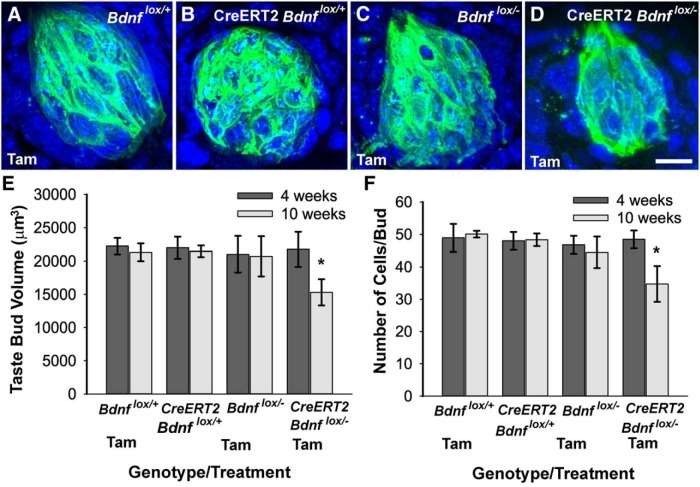
Taste bud size and cell number were reduced after *Bdnf* gene removal during adulthood. Ten weeks after tamoxifen (Tam) administration, (***A***) *Bdnf* ^lox/+^, (***B***) CreERT2 *Bdnf* ^lox/+^, and (***C***) *Bdnf* ^lox/-^ mice had normal-sized taste buds, whereas (***D***) CreERT2 *Bdnf* ^lox/+^ mice had taste buds that appeared smaller than normal. Ten weeks after tamoxifen administration, CreERT2 *Bdnf* ^lox/+^ mice showed a reduction in (***E***) taste bud volume and (***F***) number of taste cells per bud compared with *Bdnf* ^lox/+^, CreERT2 *Bdnf* ^lox/+^, and *Bdnf* ^lox/-^ mice. Scale bar: (in ***D***) ***A***–***D***, 10 µm. *p ≤ 0.05.

Because the loss of innervation during adulthood causes taste bud loss and abnormal taste bud morphology (Guth, 1957; Oakley et al., 1993), we wondered whether reduced innervation after *Bdnf* gene deletion changes the morphologic appearance of taste buds. Four weeks after tamoxifen administration, no obvious differences in taste bud size were observed among genotypes (images not shown). However, at 10 weeks, taste buds appeared smaller in mice lacking BDNF (CreERT2 *Bdnf* ^lox/-^ mice) compared with the other three genotypes (*Bdnf* ^lox/+^, CreERT2 *Bdnf* ^lox/+^, and *Bdnf* ^lox/-^; Fig. 4*A*–*D*).

To quantify this apparent reduction in taste bud size, we measured taste bud volume and cell number within taste buds. Taste buds were defined by staining with anti-cytokeratin-8 antibody, which is a marker for simple epithelium and labels many columnar taste cells of fungiform taste buds. All DAPI-stained nuclei within the cytokeratin-8 border were quantified, so that cell number was not limited to cytokeratin-8-positive taste cells. Four weeks after tamoxifen administration, there were no differences in taste bud volume or cell number among the four genotypes (Fig. 4*E*). However, 10 weeks after tamoxifen administration, mice with *Bdnf* gene deletion (CreERT2 *Bdnf* ^lox/-^ mice) showed a 30% reduction in taste bud volume compared with the other three genotypes (*Bdnf* ^lox/+^, *p* < 0.01; CreERT2 *Bdnf* ^lox/+^, *p*<0.01; *Bdnf* ^lox/-^, *p*<0.05; Fig. 4*E*). Consistent with changes in taste bud volume, CreERT2 *Bdnf* ^lox/-^ mice showed an approximately 30% reduction in the number of taste cells per bud compared with the other three genotypes 10 weeks after tamoxifen administration (*Bdnf* ^lox/+^, *p* < 0.01; CreERT2 *Bdnf* ^lox/+^, *p*<0.01; *Bdnf* ^lox/-^, *p*<0.05; Fig. 4*F*). No differences in taste bud volume or cell number were observed among the three control genotypes at either 4 or 10 weeks, ruling out any effects of tamoxifen or a single *Bdnf* allele on taste bud size. Considering the taste buds of all four genotypes together, larger taste buds contained more taste cells (*r* = 0.887, *p* < 0.001, *n* = 81). In conclusion, our results suggest that BDNF is required for maintaining normal taste bud volume during adulthood and that decreases in taste bud volume after *Bdnf* gene deletion were due to taste cell loss.

**Figure 5.**
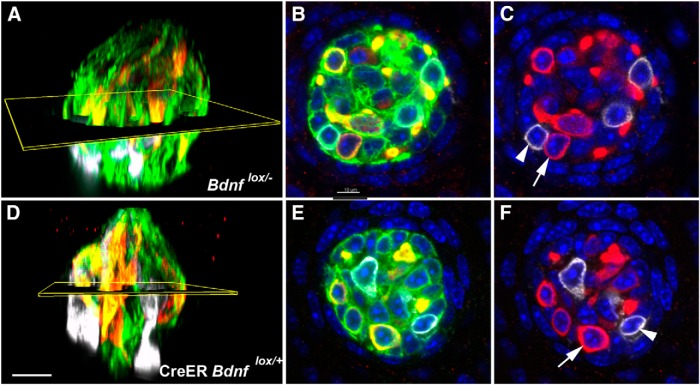
Taste receptor cells were quantified in whole taste buds from *Bdnf*l^ox/-^ (***A***) and CreER *Bdnf* ^lox/-^(***D***) mice, ten weeks after tamoxifen administration. Taste buds were imaged with the high-resolution *X*–*Y* plane in cross-section (***B*, *E***), such that individual PLCβ2-positive (red, arrow) and Car4-positive (white, arrowheads) could be quantified by following each cell from the taste pore to the basal region of the bud (***C*, *F***). Cytokeratin-8-positive cells (green) could also be quantified (***B*, *E***). Scale bar, 10µm.

Because taste bud size was reduced, we wished to determine if one particular taste receptor cell type was influenced more than others were. Taste receptor cells with the G-protein-coupled receptors for sweet, bitter, and umami were labeled with anti-PLCβ2, whereas another type that responds to acid labels with anti-Car4. Taste buds were imaged in whole mounts, so that all taste cells of a given type could be reported in whole numbers rather than percentages (Fig. 5). Because there were no differences in taste bud size in the control groups we compared one control group (*Bdnf* ^lox/-^) with the experimental group (CreERT2 *Bdnf* ^lox/-^), both of which received tamoxifen. There was no difference between genotypes in the number of PLCβ2-positive (*Bdnf* ^lox/-^ mice = 11.8 ± 0.56 vs CreERT2 *Bdnf* ^lox/-^ = 11.3 ± 1.2) or Car4-positive (*Bdnf* ^lox/-^ mice = 2.78 ± 0.55 vs CreERT2 *Bdnf* ^lox/-^ = 2.0 ± 0.38) taste receptor cells. We also labeled and quantified the cytokeratin 8 positive cells; there was a slight decrease in CreERT2 *Bdnf* ^lox/-^ (30 ± 1.03) compared with *Bdnf* ^lox/-^ (34.4 ± 1.52; *p* < 0.03). We conclude that the loss of taste cells is not due to loss of a specific taste bud cell type.

**Figure 6.**
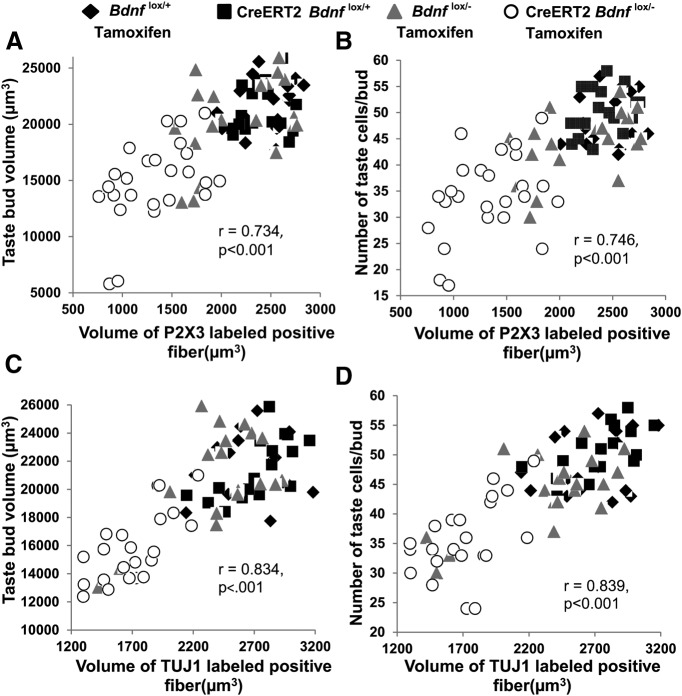
Within individual taste buds, those that lost the most innervation were also those that were smallest in size after removal of BDNF. ***A***, ***C***, Taste bud volume and (***B***, ***D***) cell number is plotted as a function of the volume of innervation.

The amount of innervation a taste bud receives during early development predicts the size of that taste bud by adulthood (Krimm and Hill, 1998a,b,2000), but it is unclear whether this relationship can be reestablished during adulthood after innervation is lost. In fact, previous studies suggest that this relationship is easily disrupted by environmental manipulations such as sodium deprivation (Krimm and Hill, 1999) and nerve regeneration (Shuler et al., 2004). Because CreERT2 *Bdnf* ^lox/-^ mice have reduced innervation to taste buds and smaller taste buds 10 weeks after tamoxifen administration, we examined whether these traits are correlated within individual taste buds. Considering the taste buds of all four genotypes together, larger taste buds and taste buds with more taste cells had greater amounts of innervation as labeled by P2X3 (*r* = 0.734, *p* < 0.001, *n* = 81; Fig. 5*A*; *r* = 0.746, *p* < 0.001, *n* = 81; Fig. 6*B*) and TUJ1 (*r* = 0.834, *p* < 0.001, *n* = 81; Fig. 5*C*; *r* = 0.839, *p* < 0.001, *n* = 81; Fig. 5*D*). In conclusion, loss of innervation was associated with smaller taste bud size and reduced cell number in CreERT2 *Bdnf* ^lox/-^ mice.

### Taste bud-derived *Bdnf* regulates innervation but not taste bud size

Because BDNF is located in the taste bud, the geniculate ganglion, and the CNS, BDNF from any of these locations could influence gustatory innervation and taste bud size. However, if BDNF is required for the innervation of new taste cells during adulthood, then taste cells should be the source of BDNF for the maintenance of innervation. To test this idea, we needed to remove BDNF from the lingual epithelium. Both taste bud cells and epithelial precursors express cytokeratins 5 and 14, suggesting that *Bdnf* could be removed from the lingual epithelium of both K5-CreERT2 and K14-CreER mice. In the K14-CreER line, recombination is induced in cells that become taste cells (Vasioukhin et al., 1999; Okubo et al., 2009), and could be used to remove *Bdnf* from the lingual epithelium without influencing *Bdnf* levels in gustatory neurons or mesenchyme.

**Figure 7.**
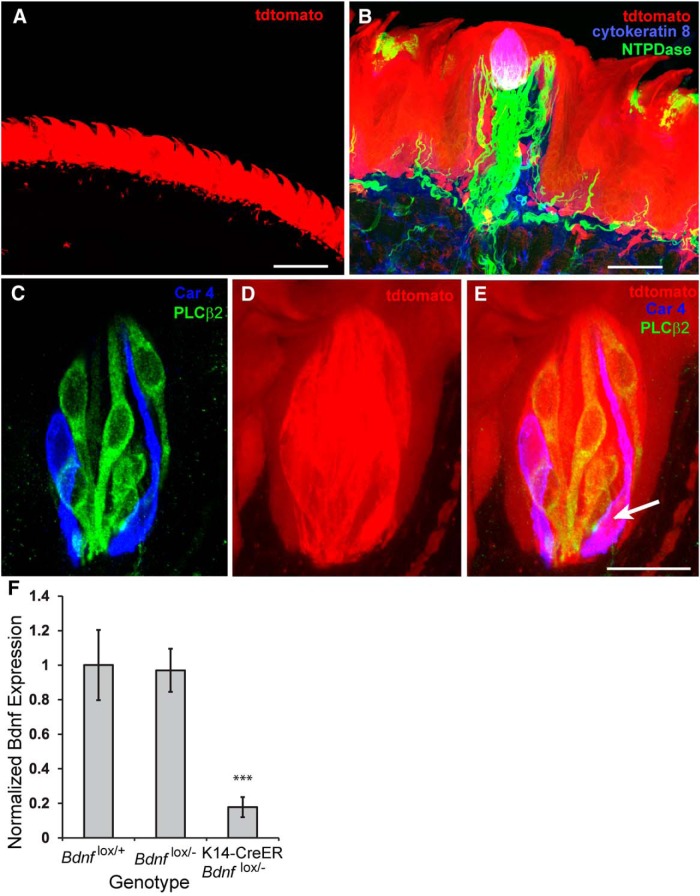
Inducible K14-CreER-mediated gene recombination was effective in lingual epithelial cells after 3 weeks of tamoxifen administration. ***A***, Ten weeks after a 3 week period of tamoxifen administration, the lingual epithelium appeared to be solidly red in K14-CreER tdtomato mice, indicating that gene recombination had occurred in lingual epithelium cells. ***B***, Tdtomato (red) with was observed in fungiform papillae. ***C***–***E***, Taste buds appeared solid red, which indicates that gene recombination occurred in many or all taste bud cells. Taste cells labeled with anti-PLCβ2 (green), which identifies cells that transduce taste via G-protein-coupled receptors for bitter, sweet, and umami (Clapp et al., 2004), and taste cells labeled with anti-Car4 (carbonic anhydrase 4; blue), which may identify cells that are responsive to sour stimuli (Chandrashekar et al., 2009), were labeled with tdtomato, indicating that they underwent gene recombination. ***E*,** arrow, The tongues used for anatomical analysis had a reduction in *Bdnf* below 20% of normal levels (***F***). These values are normalized against those for *Bdnf* ^lox/+^ mice. Scale bars: ***A***, 200 µm; ***B***, 40 µm; (in ***E***) ***C***, ***D***, ***E***, 20 µm. ***p ≤ 0.005.

To visualize the effectiveness of K14-CreER-induced gene recombination, we bred K14-CreER mice with stop-floxed tdtomato (Ai14) mice. Ten weeks after 3 weeks of tamoxifen administration, the epithelium in these mice was bright red, with few unlabeled regions (Fig. 7*A*). Fungiform papillae were completely labeled (Fig. 7*B*), and taste buds appeared to have labeling in all/most taste cells (Fig. 7*C*–*E*) including those expressing PLCβ2 and Car4, indicating that K14 progenitors contribute to both of these cell types. The tdtomato labeling was so dense that it was difficult to determine the proportion of taste cells in the buds that were labeled. However, because cells that express Car4 are the same ones that express BDNF (Huang et al., 2015), the fact that gene recombination occurred in this cell type was particularly important.

**Figure 8.**
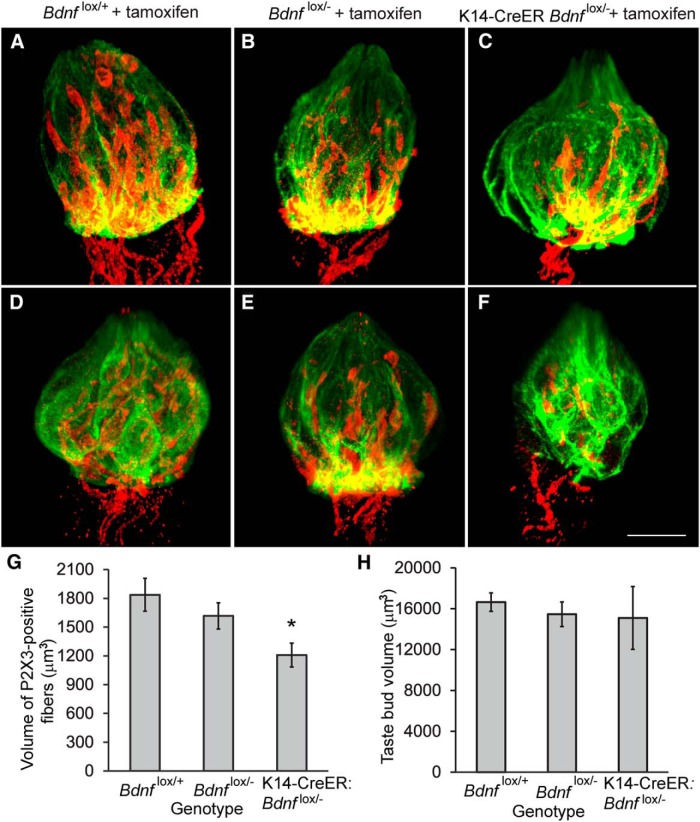
Taste buds have reduced P2X3-positive innervation after *Bdnf* reduction. ***A***–***F***, Representative whole taste buds labeled with cytokeratin-8 (green) and P2X3 (red) are shown for control (*Bdnf* ^lox/-^, *Bdnf* ^lox/+^) and experimental (K14-CreER *Bdnf* ^lox/-^) genotypes 10 weeks after tamoxifen administration. Taste buds in (***C***, ***F***) K14-CreER *Bdnf* ^lox/^mice appeared to have reduced innervation compared with (***A***, ***D***) *Bdnf* ^lox/+^ and (***B***, ***E***) *Bdnf* ^lox/-^ mice. ***F***, Some taste buds in K14-CreER *Bdnf* ^lox/-^ mice were smaller than normal, (***C***) whereas others were normal in size. ***G***, The volume of P2X3 innervation in taste buds was reduced in K14-CreER *Bdnf* ^lox/-^ mice compared with *Bdnf* ^lox/-^ and *Bdnf* ^lox/+^ mice, but (***H***) taste bud volume was not affected by *Bdnf* reduction. Scale bar, 20 µm. *p ≤ 0.05.

Based on these findings, we sought to determine whether innervation to taste buds is lost when *Bdnf* expression is reduced from the lingual epithelium by collecting tongues from eight K14-CreER *Bdnf* ^lox/-^ mice and littermate control mice (*Bdnf* ^lox/-^, *Bdnf* ^lox/+^) after 3 weeks of tamoxifen administration. One-half of the tongue epithelium was processed for RT-PCR, and the other half was processed for anatomical analysis. We found that *Bdnf* was reduced to <20% of normal levels (Fig. 7*F*; *p* < 0.005). For anatomical analysis, tongue halves were processed for thick section immunohistochemistry (70 µm) for anti-cytokeratin-8 and anti-P2X3, allowing us to capture entire taste buds in a single confocal file. P2X3-positive innervation appeared to be consistently decreased in taste buds from K14-CreER *Bdnf* ^lox/-^ mice (Fig. 7*C*,*F*) compared to littermate controls (*Bdnf* ^lox/-^, *Bdnf* ^lox/+^; Fig. 8*A*,*B*,*D*,*E*). Taste buds appeared to be variable in size, with some that were smaller than normal (Fig. 8*F*) and others that were normal in size (Fig. 8*C*). To quantify these observations, we measured taste bud volume and P2X3-positive innervation volume in 3D confocal images. We found that P2X3-positive innervation to taste buds was decreased in K14-CreER *Bdnf* ^lox/-^ mice compared with *Bdnf* ^lox/-^ and *Bdnf* ^lox/+^ control mice (*p* < 0.03; Fig. 8*G*). However, taste bud size was similar across genotypes (Fig. 8*H*). Therefore, we conclude that taste buds produce BDNF in adult mice and that this source of BDNF regulates innervation to taste buds during adulthood, but not taste bud size.

**Figure 9.**
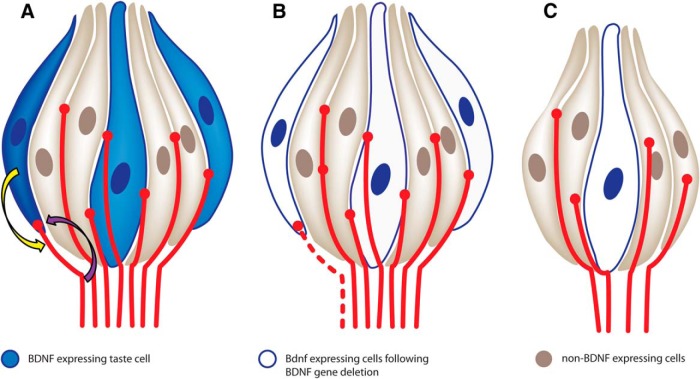
Diagram illustrating a possible role of BDNF during adulthood. ***A***, Some taste bud cells express BDNF (blue), whereas others do not (tan). BDNF in new taste cells (dark blue) attracts a subset of geniculate afferents (yellow arrow), which form functional connections with these taste cells. Taste neurons likely release some factor that maintains at least some taste cells (purple arrow). ***B***, When new taste cells no longer express BDNF, they fail to attract new innervation (dashed fiber); however, mature taste cells no longer expressing BDNF that have already been innervated are unaffected. ***C***, Given sufficient time following BDNF gene deletion, all BDNF-expressing taste receptor cells are replaced with cells that no longer express BDNF. As a result, innervation is reduced, and this loss of innervation results in a reduction in taste bud size and cell number.

## Discussion

Adult taste bud cells die and are replaced, requiring nerve fibers to continuously locate and innervate new taste cells (Beidler and Smallman, 1965; Perea-Martinez et al., 2013). This means that the maintenance of innervation is an active process such that nerve fibers that fail to locate and form connections with new taste receptor cells will eventually not be innervating taste buds. The mechanism for this process is unclear, but similar processes during initial development can provide clues. Embryonically, BDNF attracts gustatory axons to developing taste epithelium (Ringstedt et al., 1999; Lopez and Krimm, 2006b; Ma et al., 2009), and BDNF regulates synapse formation in the CNS during adulthood (Aguado et al., 2003; Cohen-Cory et al., 2010). Therefore, we speculated that BDNF, which is expressed specifically in taste receptor cells by adulthood (Yee et al., 2003), is required to maintain innervation to taste buds during the active process of taste cell renewal. To determine whether this was the case we inducibly removed *Bdnf* during adulthood. We found that taste buds lose 40% of their innervation after adult *Bdnf* gene deletion. *Bdnf* gene deletion also reduced taste bud size and cell number. Furthermore, reduction of *Bdnf* specifically in the lingual epithelium revealed that the taste bud was the primary source of BDNF controlling their innervation. Also, as *Bdnf* gene deletion had a greater effect on innervation than on taste bud size, the primary action of BDNF is likely on nerve fibers. These data demonstrate that taste-bud derived BDNF is required for the maintenance of normal levels of innervation during adulthood.

Because *Bdnf* gene deletion may be imperfect with some cells not undergoing gene recombination, the experimental animals used in this study had one null allele for *Bdnf* and one floxed allele. With our strategy, any given cell that undergoes successful *Bdnf* gene recombination is no longer capable of producing either *Bdnf* mRNA or protein. Heterozygous *Bdnf* knockouts do not have much of a reduction in *Bdnf* mRNA nor is there much effect on innervation and taste bud size, indicating that one functional allele is sufficient to produce normal levels of BDNF. Therefore, any cell that does not undergo gene recombination is likely producing normal levels of BDNF. Our RT-PCR results are an indication of the relative number of cells that successfully underwent gene recombination. Because *Bdnf* mRNA levels were barely detectable in CreERT2 *Bdnf* ^lox/-^ mice and substantially reduced in K14-CreER *Bdnf* ^lox/-^ mice, it seems clear that most of the cells in the lingual epithelium that would normally produce BDNF are no longer doing so in these animals. If BDNF production is required for these cells to be innervated, fewer cells should remain innervated following BDNF gene recombination. Therefore, even if *Bdnf* gene recombination did not occur in all taste cells, we predicted a reduction in innervation. Our data are consistent with this prediction.

We did not measure a reduction in innervation to taste buds until between 4–10 weeks after tamoxifen administration. Why did it take so long to observe an anatomical effect? If BDNF protein degrades more slowly than *Bdnf* mRNA, then the remaining low levels of BDNF could support taste innervation temporarily after *Bdnf* gene recombination, making the precise onset of BDNF removal unclear. However, it seems unlikely that this alone could account for such a substantial delay. Another possibility is the BDNF is not required to maintain innervation to taste cells that are already innervated. Instead, BDNF may support the formation of new taste receptor cell–nerve fiber connections. Loss of innervation specifically to new taste cells would take much longer than loss of innervation to existing taste cells because a sufficient number of new taste receptor cells that lack BDNF would need to be added to taste buds before a loss of innervation is observed. Different types of taste receptor cells are added to taste buds at different rates (Perea-Martinez et al., 2013). Because BDNF colocalizes with Snap-25, it is expressed in the taste receptor cell population with the slowest turnover (Yee et al., 2002; Perea-Martinez et al., 2013). Therefore, we would expect a slow loss of innervation if BDNF encourages innervation of new taste receptor cells. For this reason, we speculate that BDNF functions to regulate innervation to new taste receptor cells rather than maintaining innervation to existing ones.

Innervation to taste buds (60%) remained after *Bdnf* gene deletion. Although a few BDNF-positive taste cells may still express BDNF and be innervated, it is unlikely that this could account for all the remaining innervation. Some taste receptor cells do not express BDNF in adulthood (Yee et al., 2003), some adult gustatory neurons do not express *TrkB,* the primary receptor for BDNF (Cho and Farbman, 1999; Matsumoto et al., 2001; Farbman et al., 2004), and some taste neurons are not dependent on TrkB during development (Fei and Krimm, 2013). Therefore, most of the nerve fibers remaining after *Bdnf* removal may be a separate TrkB-negative subpopulation that innervates BDNF-negative taste bud cells. This raises the intriguing possibility that BDNF coordinates innervation of specific taste cell types (ie, BDNF-expressing) with a specific neuron type (ie, TrkB-expressing). Such a mechanism coordinating taste receptor cell types with neuron types would explain why taste neurons appear to respond to specific gustatory stimuli (Yoshida et al., 2006; Yoshida and Ninomiya, 2010; Barretto et al., 2015) even though multiple taste cells likely converge onto a single neuron. If BDNF only encourages innervation of a limited number of taste receptor cells, other mechanisms must coordinate innervation of non-BDNF expressing taste receptor cells with non-TrkB-expressing nerve fibers. Other neurotrophins expressed in adult taste cells (Takeda et al., 2004, 2005; Suzuki et al., 2007) may serve this function.

We found that BDNF maintains peripheral sensory innervation but not neuron survival in adulthood. Consistently, *in vitro* and *in vivo* studies demonstrate that adult sensory neurons do not depend on neurotrophins for survival to the same extent as they do in development (Angeletti et al., 1971; Goedert et al., 1978; Easton et al., 1997). Similarly, embryonic geniculate ganglia require exogenous neurotrophins to prevent deterioration *in vitro*, whereas adult geniculate ganglia do not (Hoshino et al., 2010). This change in neurotrophic factor dependency for many adult neurons occurs because developmental neurotrophin deprivation activates translocation of the proapoptotic molecule Bax resulting in cytochrome c release from mitochondria causing cell death; however, in adult neurons neurotrophin deprivation does not activate this pathway (Putcha et al., 2000). Although neurotrophins do not support survival in adulthood, they do support normal cell size (Angeletti et al., 1971), and we did find that geniculate neurons were smaller following BDNF removal. However, the effect was not nearly as dramatic as loss of NGF on sympathetic neurons in adulthood (Angeletti et al., 1971). This difference could be due to differences in sympathetic and sensory ganglion or perhaps some geniculate neurons lose sensitivity to BDNF by adulthood (Cho and Farbman, 1999; Matsumoto et al., 2001; Farbman et al., 2004) and become dependent on another factor. Alternatively, this could be a difference in the adult roles of BDNF compared with NGF. Such that BDNF regulation of peripheral sensory neurons in adulthood may be similar to the postnatal and adult roles of BDNF in the central nervous system, where BDNF also regulates dendritic morphology and connectivity rather than maintaining number and cell body size (Gorski et al., 2003a; Rauskolb et al., 2010; Hiester et al., 2013).

Our finding that BDNF maintains sensory innervation to taste buds is consistent with correlative studies in multiple sensory systems (Bergman et al., 2000; Gardiner et al., 2008; Ola et al., 2013). Specifically, with aging, decreases in NT3 and NT4 accompany the loss of sensory axons into peripheral receptive fields (Bergman et al., 1999, 2000). Sensory nerve fiber loss in sensory neuropathies is accompanied by reduced neurotrophins (Anand, 2004). Also, the loss of sensory innervation seen in diabetic neuropathies is rescued by the addition of neurotropic factors (Christianson et al., 2003, 2007). In several neurodegenerative diseases, fungiform papillae number or taste sensation are reduced, and this is accompanied by a reduction of neurotrophins (Gardiner et al., 2008). However, these previous studies are correlative; although both neurotrophins and sensory innervation are reduced, a causal relationship between the loss of neurotrophins in adulthood and the loss of innervation was not established. Here, we confirmed that reduction of neurotrophins causes the loss of sensory innervation.

We found a greater loss of innervation when gene recombination was under control of the ubiquitin promoter than with the K14-CreER construct, probably due to differences in the effectiveness of *Bdnf* gene recombination between the two mouse lines. Differences in the number of cells that underwent gene recombination can also explain why taste bud size and cell number were reduced in CreER *Bdnf* ^lox/-^ mice but not in K14-CreER *Bdnf* ^lox/-^ mice. Consistently, *Bdnf* gene deletion had a greater impact on innervation than on taste bud size/number in CreER *Bdnf* ^lox/-^ mice, indicating that innervation is more sensitive to *Bdnf* removal. It is possible that if *Bdnf* expression levels were reduced to 5% of normal levels in K14-CreER *Bdnf^lox/lox^* mice (indicating that just as many cells underwent *Bdnf* gene deletion) then taste bud size would have also been reduced. However, it is also possible that BDNF from a source other than taste buds maintains taste bud size. Lastly, it is possible that the impact on taste bud size in CreER *Bdnf* ^lox/-^ mice is due to an indirect effect (i.e., obesity). Consistent with the idea of an indirect or nonspecific effect of BDNF removal of taste bud size, taste cell loss was not specific to the Car4-positive taste receptor cells known to express BDNF (Yee et al., 2003; Huang et al., 2015), and instead appears to be due to a small reduction in all cell types.

Our findings suggest the reinterpretation of an earlier study in which BDNF overexpression in α-gustducin-expressing taste receptor cells slightly increased innervation to taste buds (Nosrat et al., 2012). BDNF overexpression likely begins at birth in these mice (Ohtubo et al., 2012), and because remodeling likely occurs during postnatal peripheral gustatory development (Nagai et al., 1988; Kinnamon et al., 2005; Huang et al., 2015), BDNF overexpression under the control of a gustducin promoter could increase innervation via multiple mechanisms, including prevention of postnatal remodeling (Huang et al., 2015). However, because α-gustducin-expressing taste cells do not normally express BDNF (Yee et al., 2003), BDNF expressed in these taste receptor cells could have attracted abnormal amounts or types of innervation in adulthood. If this were the case, these mice might be expected to have increased responsiveness to sweet or bitter stimuli. Unfortunately, this was not tested in these mice, but another BDNF-overexpressing mouse line demonstrated increased responses to sweet and the mechanisms may be similar (Sun et al., 2015).

This is the first study to examine the effects of BDNF removal from adult taste buds, these findings plus what we already know concerning normal BDNF expression in the adult taste system allowed us to develop the following testable model concerning the potential role of BDNF in adulthood (Fig. 8). BDNF is expressed in some adult taste cells but not others (Yee et al., 2003; Huang et al., 2015). During taste cell turnover, BDNF expressed in new taste receptor cells recruits innervation and stimulates the formation of functional connections between taste receptor cells and nerve fibers. When BDNF is removed during adulthood, new BDNF-expressing taste cells no longer recruit innervation, so these cells remain uninnervated. Because innervation is required to support normal morphology and taste bud cell number (Guth, 1957; Oakley et al., 1993; Guagliardo and Hill, 2007), the number of taste cells within buds are reduced. However, innervation to non-BDNF expressing taste cells remains unaffected. This model provides the first potential mechanism to explain how nerve fibers could connect to a continuously renewing population of taste cells and yet maintain a constant neural code.

## Synthesis

The decision was a result of the Reviewing Editor Piali Sengupta and the peer reviewers coming together and discussing their recommendations until a consensus was reached. A fact-based synthesis statement explaining their decision and outlining what is needed to prepare a revision is listed below. The following reviewers agreed to reveal their identity: Linda Barlow, Brian Pierchala

Both reviewers were very positive about the significance and methodology of the work. They only had a couple of major and a few minor comments that will need to be addressed prior to a formal acceptance.

Main comments:

1. Is the reduced taste bud size primarily due to loss of Car4+ cells and/or other taste cell types?

2. Please determine whether loss of BDNF innervation correlates with somal atrophy. Lack of atrophy may indicate an alternate source of trophic innervation.

Minor comments:

1. There is a sentence structure problem in the third sentence of the Significance Statement.

2. The Huang et. al. 2015 reference is duplicated in the References list, and is incomplete.

3. Sentence 4 of the results is difficult to understand.

4. On page 17 in the results, the authors describe in detail experiments in which they compared the K5-ERT2 line with the K14-CreER line in regards to Bdnf deletion, but they do not show any of the data. I recommend either showing the data, or removing this paragraph entirely. It is not necessary given that Figure 6 shows clearly the effectiveness of the K14-CreER line.

5. In the Results section describing Figure 6, the BDNF expression data is panel F, not H, as described in the text. In addition, panel F is not in the text of the Figure legend with its description.

6. The Putcha et. al. 2000 reference is not in the References list.

7. The Putcha et. al. 2000 study is perhaps not the best reference to site for the concept of neuronal maturation. Very early studies, in fact, demonstrated in vivo the loss of trophic factor dependence for survival (Angeletti et. al., Brain Res., 1971; Goedert et. al., Brain Res., 1978). And the first study showing this in culture was Easton et. al., J. Neurosci, 1997. The authors should consider including these and other studies in the area of neuronal maturation in their discussion.

8. p18 - "deprivation" and not "derivation"

9. The Gardiner et al 2008 Neuroscientist reference is missing.
